# Supplementary material for: Development and Feasibility of an Inpatient Cancer-Related Sarcopenia Pathway at a Major Cancer Centre
Source: Int J Environ Res Public Health. 2022 Mar 29;19(7):4038. doi: 10.3390/ijerph19074038 (PMC8997788; doi:10.3390/ijerph19074038)
Supplement: Supplementary file 1 [file ijerph-19-04038-s001.zip › Supplementary File S1. The sarcopenia management pathway (sarc-pathway) model.pdf]

THE SARC-PATHWAY

SCREENING

AHA-NA to complete sarcopenia screening within 48hrs of admission, provide education material to all patients *(NB. to measure weight, height, calculate BMI and complete MST if not yet completed)*

Repeat screening at day 7 of admission (+/- 1 day)

1. HGS[2]: <27kg (males), <16kg (females) [document 3x attempts (bilaterally) & max. result]
2. SARC-CalF: ≥11 [document result for each component of SARC-F, 2x calf circumference (CC) bilaterally & max.

YES – if meets cut-offs for HGS AND/OR SARC-CalF

NO

AT RISK OF SARCOPENIA (or PROBABLE SARCOPENIA)

NOT AT RISK of sarcopenia

Referral to dietitian (DT) by AHA-NA in EMR (even if DT already involved in care)

Referral to physiotherapist (PT) by AHA-NA in EMR (even if PT already involved in care)

ASSESSMENT

DT assessment within 24hrs of referral, including full nutrition assessment of medical, social, biochemical, nutrition impact symptoms, PG-SGA, BIS via SOZO [document skeletal muscle mass 4x limbs, fat-free mass; calculate ALM] & clinical interpretation and consideration of symptom management/referral to MDT as appropriate.

PT assessment within 24hrs of referral, including 5-CST [document time taken], AKPS and consideration of symptom management/referral to MDT as appropriate.

LOW MUSCLE STRENGTH = HGS: <27kg (males), <16kg (females) **AND/OR** 5-CST: >15secs
AND
LOW MUSCLE MASS = ALM: <20kg (males), <15 (females) [if not available, use BMI-adjusted CC as proxy[3]]

YES

NO

PATIENT DIAGNOSED AS HAVING SARCOPENIA
(DT to document in EMR)

Patient remains AT RISK OF SARCOPENIA (or PROBABLE SARCOPENIA)

TREATMENT

Patient requires tailored, multidisciplinary nutrition and exercise care

DT to provide individualised medical nutrition therapy including[4]:

- Dietary counselling on preservation/ ↑ lean muscle
- Minimum 105-125kJ/kg/day energy & 1-1.5g/kg/day protein (ONS +/- EN/PN)
- Food service assistance
- Relevant education material
- Frequency of review based on individual needs
- Repeat weekly measures of PG-SGA, HGS, BIS and within 1-2 days of hospital discharge (min. 5 days since last measurements)

PT to provide individualised exercise prescription including[4]:

- Targeted resistance +/- aerobic exercise program
- Referral to exercise class
- Relevant education material
- Frequency of review based on individual needs
- +/- referral to AHA-PT to encourage/assist with PT's exercise prescription
- Complete 5-CST at each attendance (including within 1-2 days of hospital discharge)

If ongoing care provided post-discharge, DT and/or PT to see in outpatients and/or refer to appropriate community provider

MULTIDISCIPLINARY COLLABORATIVE CARE

KEY:

5-CST = 5 times chair stand test
AHA-NA = allied health assistant - nutrition assistant
AHA-PT = allied health assistant - physiotherapy
AKPS = Australian-modified Karnofsky Performance Status
ALM = appendicular lean mass
BIS = bioimpedance spectroscopy
BMI = body mass index
CC = calf circumference
DT = dietitian
EMR = electronic medical record
EN = enteral nutrition
HGS = hand grip strength
MDT = multidisciplinary team including medical, nursing, allied health professionals, other support staff
MST = Malnutrition Screening Tool
ONS = oral nutrition support
PG-SGA = Patient-Generated Subjective Global Assessment
PN = parenteral nutrition
PT = physiotherapist
SARC-CalF = 5-item sarcopenia screening tool combined with calf circumference measurement with scoring system
SARC-F = 5-item screening tool for sarcopenia
SOZO = SOZO® Digital Health Platform (bioimpedance spectroscopy device)

FOOTNOTES:

[1] Refer to relevant Sarc-pathway training module

[2] 5-CST as a proxy for muscle strength if HGS not available:
Cut-offs: Low muscle strength = >15 seconds to complete 5x chair stands

[3] BMI-adjusted calf circumference as a proxy for muscle mass if ALM not available:
Cut-offs: Low muscle mass = if BMI 18.5-24.9kg/m^2: Males: ≤34cm Females: ≤33cm

+ BMI adjustment if BMI outside 18.5-24.9kg/m^2 (same for both males and females):

BMI group (kg/m^2)	Adjustment factor, cm (rounded)
<18.5	+4.0
25-29.9	-3.0
30-39.9	-7.0
≥40	-12.0

[4] If patient does not require ongoing DT and PT interventions whilst an inpatient, refer back to AHA-NA for rescreening
