# Supplementary material for: Development and Feasibility of an Inpatient Cancer-Related Sarcopenia Pathway at a Major Cancer Centre
Source: Int J Environ Res Public Health. 2022 Mar 29;19(7):4038. doi: 10.3390/ijerph19074038 (PMC8997788; doi:10.3390/ijerph19074038)
Supplement: Supplementary file 1 [file ijerph-19-04038-s001.zip › Supplementary File S2. Participant & clinician acceptability survey.pdf]

**Supplementary File S2.** Participant and clinician acceptability survey of the care on the sarc-pathway.

## A. Participant acceptability survey of the sarc-pathway

### Survey questions

1. How old are you?
   ______________ years

2. Are you:
   ☐ Male
   ☐ Female
   ☐ Prefer not to say
   ☐ Other (please specify:________________________________________________________)

3. What is your postcode?
   __________________________

4. Is English your first language?
   ☐ Yes
   ☐ No, (please specify first language: ______________________________________________)

5. Are you of Aboriginal or Torres Strait Islander origin?
   ☐ Yes: ☐Aboriginal
   ☐Torres Strait Islander
   ☐Both Aboriginal and Torres Strait Islander
   ☐ No
   ☐ Prefer not to say

6. Which of the below best describes your cancer diagnosis?
   ☐ Lung
   ☐ Breast
   ☐ Genitourinary oncology (kidney, bladder, prostate, testicles, penis)
   ☐ Head and neck (mouth, nose, throat, voice box)
   ☐ Melanoma and skin
   ☐ Neuro-oncology (brain and spine)
   ☐ Upper gastrointestinal (upper section of the gastrointestinal tract)
   ☐Lower gastrointestinal (colorectal, bowel, colon)
   ☐ Haematology (blood)
   ☐ Gynae-oncology (uterus, ovaries, fallopian tubes, cervix, vagina, vulva)
   ☐ Sarcoma (bone, muscle, nerves, other soft tissues that connect/surround joints)
   ☐ Unknown primary
   ☐Other (please specify cancer diagnosis: ________________________________________)

7. At which time-point are you currently at in your cancer journey? Tick the one that most closely applies.
   ☐ I have just been diagnosed with cancer
   ☐ I am waiting for treatment
   ☐ I am receiving cancer treatment
   ☐ Treatment has finished: What year did you finish treatment? ___________________________
   ☐ I have been diagnosed with cancer but did not receive treatment
   ☐ Palliative care/end of life

☐ Other (please specify time-point: ______________________________________________)

8. Which cancer treatment(s) are you receiving or have you received? (Select all that apply)
   ☐ Surgery
   ☐ Chemotherapy: ☐ Oral
   ☐ Intravenous (drip)
   ☐ Radiotherapy
   ☐ Stem cell transplant
   ☐ Immunotherapy
   ☐ Hormone therapy
   ☐ Other (please specify cancer treatment:___________________________________________)

9. This question relates to the session you had with the NUTRITION ASSISTANT when you were admitted to the ward. The NUTRITION ASSISTANT asked you some questions to see if you were 'at risk' of sarcopenia. Please note below if you agree or disagree with the following statements or tick N/A if this is not applicable to you.

| | N/A | Strongly disagree | Disagree | Slightly agree | Agree | Strongly agree |
|---|---|---|---|---|---|---|
| Provided an element of enjoyment (i.e. it was engaging) | ☐ | ☐ | ☐ | ☐ | ☐ | ☐ |
| Took too much time or effort | ☐ | ☐ | ☐ | ☐ | ☐ | ☐ |
| Was completed in comfort and fits with my personal values | ☐ | ☐ | ☐ | ☐ | ☐ | ☐ |
| Was easily understood and made sense to me | ☐ | ☐ | ☐ | ☐ | ☐ | ☐ |
| I saw personal benefit in this session | ☐ | ☐ | ☐ | ☐ | ☐ | ☐ |
| It helped me know if I was 'at risk' of sarcopenia or not (i.e. achieved its purpose) | ☐ | ☐ | ☐ | ☐ | ☐ | ☐ |
| I felt confident that the NUTRITION ASSISTANT came up with a good plan for helping me i.e. arranged for me to be seen by the Dietitian, Physiotherapist or Exercise Physiologist | ☐ | ☐ | ☐ | ☐ | ☐ | ☐ |
| I was overall satisfied with the session that the NUTRITION ASSISTANT completed | ☐ | ☐ | ☐ | ☐ | ☐ | ☐ |

***If you were NOT 'at risk' of sarcopenia and did not go onto see a dietitian, physiotherapist or exercise physiologist, please go to question 12 (otherwise continue onto question 10.)***

**10.** This question relates to the sessions you may have had with the DIETITIAN and/or PHYSIOTHERAPIST and/or EXERCISE PHYSIOLOGIST on the ward or in the gym. They will have asked you some questions, may have done some tests to check your strength, given you diet and/or exercise advice and/or you may have attended an exercise class in the gym.

a. Did you see the DIETITIAN?
☐ Yes
☐ No
☐ Unsure

b. Did you see the PHYSIOTHERAPIST?
☐ Yes
☐ No
☐ Unsure

c. Did you see the EXERCISE PHYSIOLOGIST?
☐ Yes – on the ward
☐ Yes – I attended exercise class in the gym
☐ No
☐ Unsure

d. This section asks you to think about your overall experience of the sessions you may have had with the DIETITIAN and/or PHYSIOTHERAPIST and/or EXERCISE PHYSIOLOGIST. Please note below if you agree or disagree with the following statements or tick N/A if this is not applicable to you.

| | N/A | Strongly disagree | Disagree | Slightly agree | Agree | Strongly agree |
|---|---|---|---|---|---|---|
| Provided an element of enjoyment (engaging) | ☐ | ☐ | ☐ | ☐ | ☐ | ☐ |
| Took too much time or effort | ☐ | ☐ | ☐ | ☐ | ☐ | ☐ |
| Was completed in comfort and fits with my personal values | ☐ | ☐ | ☐ | ☐ | ☐ | ☐ |
| Was easily understood and made sense to me | ☐ | ☐ | ☐ | ☐ | ☐ | ☐ |
| I saw personal benefit in seeing the DIETITIAN, PHYSIOTHERAPIST AND/OR EXERCISE PHYSIOLOGIST | ☐ | ☐ | ☐ | ☐ | ☐ | ☐ |
| It helped me know what I had to do to improve my health in relation to being 'at risk' of sarcopenia | ☐ | ☐ | ☐ | ☐ | ☐ | ☐ |
| I felt confident that the DIETITIAN, PHYSIOTHERAPIST AND/OR EXERCISE PHYSIOLOGIST came up with a good plan for helping me i.e. diet and exercise advice/plan | ☐ | ☐ | ☐ | ☐ | ☐ | ☐ |
| I was overall satisfied with the DIETITIAN, PHYSIOTHERAPIST AND/OR EXERCISE | ☐ | ☐ | ☐ | ☐ | ☐ | ☐ |

| PHYSIOLOGIST's sessions | |
|---|---|

**11.** Do you have specific comments about your sessions with the DIETITIAN, PHYSIOTHERAPIST and/or EXERCISE PHYSIOLOGIST on the ward?

Comments: ______________________________________________

**12.** Do you have any further comments?

Comments: ______________________________________________

---

**<u>B. Clinician acceptability survey of the sarc-pathway</u>**

**Survey Questions**

1. What is your professional discipline?

☐Dietitian
☐Physiotherapist
☐Exercise physiologist
☐Nutrition Assistant

2. How many years have you worked in this profession?
☐Less than 1 year
☐1 to 5 years
☐6 to 10 years
☐11 to 20 years
☐More than 20 years

**<u>Acceptability of the sarcopenia pathway</u>**

If you are a Nutrition Assistant please complete *question 3*.

If you are a Dietitian please complete *question 4*.

If you are a Physiotherapist please complete *question 5*.

If you are an Exercise physiologist please complete *question 6*.

3. In relation to the SCREENING COMPONENT of the Sarcopenia Pathway performed by you as the *Nutrition Assistant*, please indicate if you agree or disagree with the following statements.

| **SCREENING COMPONENT** of the Sarcopenia Pathway | Strongly disagree | Disagree | Slightly agree | Agree | Strongly agree |
|---|---|---|---|---|---|
| Completing the screening component provided an element of enjoyment (i.e. it was engaging) | ☐ | ☐ | ☐ | ☐ | ☐ |
| Took too much time or effort | ☐ | ☐ | ☐ | ☐ | ☐ |
| Fits with my personal values | ☐ | ☐ | ☐ | ☐ | ☐ |
| Was easy to conduct and made sense to me | ☐ | ☐ | ☐ | ☐ | ☐ |
| I saw personal benefit in undertaking screening for sarcopenia on patients | ☐ | ☐ | ☐ | ☐ | ☐ |
| I felt confident in conducting the sarcopenia screening component | ☐ | ☐ | ☐ | ☐ | ☐ |
| It helped to identify patients 'at risk' of sarcopenia (i.e. achieved its purpose) | ☐ | ☐ | ☐ | ☐ | ☐ |
| I felt confident that the result of screening helped to come up with a good plan for the patient i.e. referral to the dietitian or physiotherapist | ☐ | ☐ | ☐ | ☐ | ☐ |
| The introduction of the Sarcopenia Pathway into clinical care improves the screening, assessment, diagnosis and intervention of sarcopenia for patients | ☐ | ☐ | ☐ | ☐ | ☐ |
| The introduction of the Sarcopenia Pathway into clinical care improves patient outcomes and care | ☐ | ☐ | ☐ | ☐ | ☐ |

*Go to question 7.*

4. In relation to the ASSESSMENT, DIAGNOSIS AND INTERVENTION COMPONENT of the Sarcopenia Pathway performed by you as the _Dietitian_, please indicate if you agree or disagree with the following statements.

| **ASSESSMENT, DIAGNOSIS AND INTERVENTION COMPONENT** of the Sarcopenia Pathway | Strongly disagree | Disagree | Slightly agree | Agree | Strongly agree |
|---|---|---|---|---|---|
| Completing the assessment, diagnosis and intervention component provided an element of enjoyment (i.e. it was engaging) | ☐ | ☐ | ☐ | ☐ | ☐ |
| Took too much time or effort | ☐ | ☐ | ☐ | ☐ | ☐ |
| Fits with my personal values | ☐ | ☐ | ☐ | ☐ | ☐ |
| Was easy to conduct and made sense to me | ☐ | ☐ | ☐ | ☐ | ☐ |
| I saw personal benefit in undertaking assessment, diagnosis and intervention for sarcopenia on patients | ☐ | ☐ | ☐ | ☐ | ☐ |
| I felt confident in conducting the sarcopenia assessment, diagnosis and intervention component | ☐ | ☐ | ☐ | ☐ | ☐ |
| It helped to assess patients 'at risk' of sarcopenia and determine an appropriate diagnosis and intervention tailored to them (i.e. achieved its purpose) | ☐ | ☐ | ☐ | ☐ | ☐ |
| I felt confident that I helped to come up with a good plan for the patient i.e. interventions, goals, education | ☐ | ☐ | ☐ | ☐ | ☐ |
| The introduction of the Sarcopenia Pathway into clinical care improves the screening, assessment, diagnosis and intervention of sarcopenia for patients | ☐ | ☐ | ☐ | ☐ | ☐ |
| The introduction of the Sarcopenia Pathway into clinical care improves patient outcomes and care | ☐ | ☐ | ☐ | ☐ | ☐ |

*Go to question 7.*

5. In relation to the ASSESSMENT, DIAGNOSIS AND INTERVENTION COMPONENT of the Sarcopenia Pathway performed by you as the *Physiotherapist*, please indicate if you agree or disagree with the following statements.

| **ASSESSMENT, DIAGNOSIS AND INTERVENTION COMPONENT** of the Sarcopenia Pathway | Strongly disagree | Disagree | Slightly agree | Agree | Strongly agree |
|---|---|---|---|---|---|
| Completing the assessment, diagnosis and intervention component provided an element of enjoyment (i.e. it was engaging) | ☐ | ☐ | ☐ | ☐ | ☐ |
| Took too much time or effort | ☐ | ☐ | ☐ | ☐ | ☐ |
| Fits with my personal values | ☐ | ☐ | ☐ | ☐ | ☐ |
| Was easy to conduct and made sense to me | ☐ | ☐ | ☐ | ☐ | ☐ |
| I saw personal benefit in undertaking assessment, diagnosis and intervention for sarcopenia on patients | ☐ | ☐ | ☐ | ☐ | ☐ |
| I felt confident in conducting the sarcopenia assessment, diagnosis and intervention component | ☐ | ☐ | ☐ | ☐ | ☐ |
| It helped to assess patients 'at risk' of sarcopenia and determine an appropriate diagnosis and intervention tailored to them (i.e. achieved its purpose) | ☐ | ☐ | ☐ | ☐ | ☐ |
| I felt confident that I helped to come up with a good plan for the patient i.e. interventions, goals, education | ☐ | ☐ | ☐ | ☐ | ☐ |
| The introduction of the Sarcopenia Pathway into clinical care improves the screening, assessment, diagnosis and intervention of sarcopenia for patients | ☐ | ☐ | ☐ | ☐ | ☐ |
| The introduction of the Sarcopenia Pathway into clinical care improves patient outcomes and care | ☐ | ☐ | ☐ | ☐ | ☐ |

*Go to question 7.*

6. In relation to the ASSESSMENT, DIAGNOSIS AND INTERVENTION COMPONENT of the Sarcopenia Pathway performed by you as the *Exercise Physiologist*, please indicate if you agree or disagree with the following statements.

| **ASSESSMENT, DIAGNOSIS AND INTERVENTION COMPONENT** of the Sarcopenia Pathway | Strongly disagree | Disagree | Slightly agree | Agree | Strongly agree |
|---|---|---|---|---|---|
| Completing the assessment, diagnosis and intervention component provided an element of enjoyment (i.e. it was engaging) | ☐ | ☐ | ☐ | ☐ | ☐ |
| Took too much time or effort | ☐ | ☐ | ☐ | ☐ | ☐ |
| Fits with my personal values | ☐ | ☐ | ☐ | ☐ | ☐ |
| Was easy to conduct and made sense to me | ☐ | ☐ | ☐ | ☐ | ☐ |
| I saw personal benefit in undertaking assessment, diagnosis and intervention for sarcopenia on patients | ☐ | ☐ | ☐ | ☐ | ☐ |
| I felt confident in conducting the sarcopenia assessment, diagnosis and intervention component | ☐ | ☐ | ☐ | ☐ | ☐ |
| It helped to assess patients 'at risk' of sarcopenia and determine an appropriate diagnosis and intervention tailored to them (i.e. achieved its purpose) | ☐ | ☐ | ☐ | ☐ | ☐ |
| I felt confident that I helped to come up with a good plan for the patient i.e. interventions, goals, education | ☐ | ☐ | ☐ | ☐ | ☐ |
| The introduction of the Sarcopenia Pathway into clinical care improves the screening, assessment, diagnosis and intervention of sarcopenia for patients | ☐ | ☐ | ☐ | ☐ | ☐ |
| The introduction of the Sarcopenia Pathway into clinical care improves patient outcomes and care | ☐ | ☐ | ☐ | ☐ | ☐ |

*Go to question 7.*

7. Do you have any suggestions on how to improve the sarcopenia pathway?

   Comments: ______________________________________________

8. Do you have any further comments?

   Comments: ______________________________________________
