# Supplementary material for: Development and Feasibility of an Inpatient Cancer-Related Sarcopenia Pathway at a Major Cancer Centre
Source: Int J Environ Res Public Health. 2022 Mar 29;19(7):4038. doi: 10.3390/ijerph19074038 (PMC8997788; doi:10.3390/ijerph19074038)
Supplement: Supplementary file 1 [file ijerph-19-04038-s001.zip › Supplementary File S3. Adherence to best practice audit.pdf]

Supplementary File S3. Adherence audit of sarcopenia clinical practices compared to recommendations and practice tips from the Cancer-related malnutrition and sarcopenia position statement[1].

Position Statement Recommendations	Pre-pilot[2]	Post-pilot[2]
All people with cancer should be screened for sarcopenia at diagnosis and repeated as the clinical situation changes, using the validated screening tool SARC-F or SARC-F in combination with calf circumference.	Did not meet	Partially met
All people with cancer identified as being 'at risk' of sarcopenia following appropriate screening should have a comprehensive evaluation of muscle status using a combination of assessments for muscle mass, muscle strength and function.	Did not meet	Partially met
Interpretation of diagnostic criteria for sarcopenia should be applied recognising that: threshold values for assessing muscle mass, muscle strength and physical performance are variable; care should be taken to determine the appropriate cut-off values in the population in which they are being applied; most data regarding muscle strength and performance comes from older populations; the applicability of diagnostic criteria in different ethnicities is uncertain.	Did not meet	Met
All people with cancer-related sarcopenia should have access to the core components of treatment including medical nutrition therapy, targeted exercise prescription and physical activity advice, and physical and psychological symptom management.	Met	Met
Treatment for cancer-related malnutrition and sarcopenia should be individualised, in collaboration with the multidisciplinary team (MDT), and tailored to meet needs at each stage of cancer treatment.	Did not meet	Met
Health services should ensure a broad range of health care professionals across the MDT have the skills and confidence to recognise malnutrition and sarcopenia to facilitate timely referrals and treatment.	Did not meet	Met
MDTs should work towards an individualised and coordinated approach to treating cancer-related malnutrition and sarcopenia.	Did not meet	Met

Position Statement Practice Tips		Pre-pilot[2]	Post-pilot[2]
Identification	Consider incorporating screening for malnutrition and sarcopenia into existing multidisciplinary and/or supportive care screening processes or patient-reported outcomes to aid ease of completion and compliance, reduce the need for additional resources and to support the initiation of appropriate assessment and care.	Did not meet	Met

Assessment	Screening should focus on early identification using a systematised model of care or pathway that defines the tools to be used, who will conduct screening, the timing and frequency of screening, and pathways for treatment referrals appropriate to the setting.	Did not meet	Met
	Malnutrition/sarcopenia assessment should be incorporated into the appropriate nutrition care policy directives with local governance, management committees and performance review processes embedded to support successful and sustainable implementation.	Did not meet	Partially met
	A measure of muscle mass should be a component of assessment of malnutrition and sarcopenia and incorporated into routine clinical practice.	Partially met	Met
	Identification of barriers and enablers to malnutrition and sarcopenia assessment at individual, team and system levels is the first step to facilitate adherence to evidence-based nutrition care recommendations and policies.	Did not meet	Partially met
Treatment	Models of care to treat malnutrition and sarcopenia should provide consistent information regarding cancer-related malnutrition and sarcopenia across disciplines and throughout phases of treatment to ensure reinforcement of a clear treatment plan.	Did not meet	Met
Multi-disciplinary care	Consider the use of a care pathway, or similar process, developed by MDT members and people with cancer to support implementation of optimal care for cancer-related malnutrition and sarcopenia.	Did not meet	Met
	Engage consumers in the development and evaluation of MDT services across the continuum of care.	Did not meet	Partially met
	Utilise a framework, for example team mental model, to develop and refine MDT services to optimise the success of the team, and importantly clinical and patient-reported outcome and experience measures.	Did not meet	Partially met

[1] Kiss N, Loeliger J, Findlay M, et al. Clinical Oncology Society of Australia: Position statement on cancer-related malnutrition and sarcopenia. Nutr Diet. 2020 Sep;77(4):416-425. doi: 10.1111/1747-0080.12631. Epub 2020 Aug 17. [2] *Met* = occurs ≥80% of the time; *partially met* = occurs ≥50-79% of the time; *did not meet* = occurs <50% of the time.
